# Supplementary material for: Meta-Analysis of COVID-19 Metabolomics Identifies Variations in Robustness of Biomarkers
Source: Int J Mol Sci. 2023 Sep 21;24(18):14371. doi: 10.3390/ijms241814371 (PMC10531504; doi:10.3390/ijms241814371)
Supplement: Supplementary file 1 [file ijms-24-14371-s001.zip › ijms-2596037-supplementary.pdf]

Meta-Analysis of COVID-19 Metabolomics Identifies Variations in Robustness of Biomarkers

Supplementary Material

Table S1. optimised GridSearchCV parameters by panel.

Panel	C	Penalty	Solver
1. Tomo *et al* (2022) [29]	0.1	*'l2'*	liblinear
2. Fraser *et al* (2020) [30]	0.1	*'l2'*	liblinear
3. Almulla *et al* (2022) [31]	0.1	*'l2'*	liblinear
4. Khodadoust *et al* (2021) [32]	1.0	*'l2'*	liblinear
5. Caterino *et al* (2021) [33]	10	*'l2'*	liblinear
6. Crowdsourced Panel	0.1	*'l2'*	liblinear

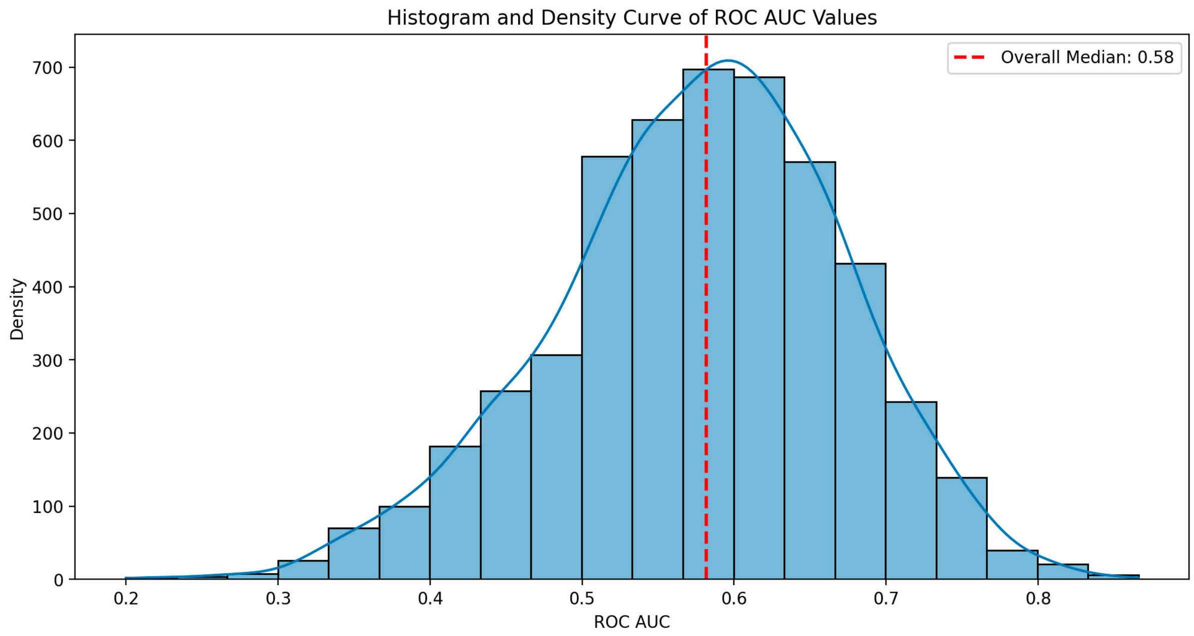

Figure S1. histogram and density plot of AUROC across 5 000 randomly generated biomarker panels, each comprising 5 metabolites selected at random from the independent dataset.

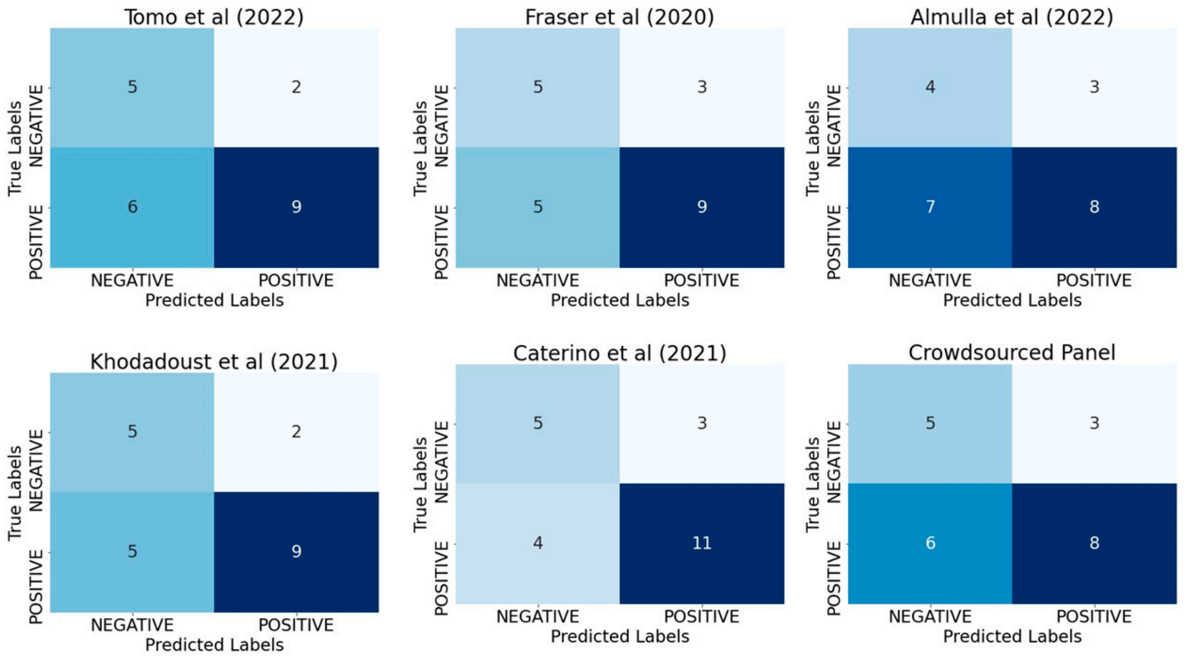

Figure S2. confusion matrices by panel.
